# CT-Derived Body Composition Values and Complications After Pneumonectomy in Lung Cancer Patients: Time for a Sex-Related Analysis?

**DOI:** 10.3389/fonc.2022.826058

**Published:** 2022-03-15

**Authors:** Stefania Rizzo, Francesco Petrella, Claudia Bardoni, Lorenzo Bramati, Andrea Cara, Shehab Mohamed, Davide Radice, Giorgio Raia, Filippo Del Grande, Lorenzo Spaggiari

**Affiliations:** ^1^ Service of Radiology, Imaging Institute of Southern Switzerland (IIMSI), Ente Ospedaliero Cantonale (EOC), Lugano, Switzerland; ^2^ Facoltà di Scienze biomediche, Università della Svizzera italiana (USI), Lugano, Switzerland; ^3^ Department of Thoracic Surgery, European Institute of Oncology (IEO), IRCCS, Milan, Italy; ^4^ Department of Oncology and Hemato-Oncology, University of Milan, Milan, Italy; ^5^ Division of Epidemiology and Biostatistics, European Institute of Oncology (IEO), IRCCS, Milan, Italy

**Keywords:** lung cancer, body composition, muscle, fat, sex, complications, pneumonectomy

## Abstract

**Purpose:**

This study aimed to assess if CT-derived body composition values and clinical characteristics are associated with the risk of postsurgical complications in men and women who underwent pneumonectomy for lung cancer.

**Materials and Methods:**

Patients who underwent pneumonectomy between 2004 and 2008 were selected. The ethics committee approved this retrospective study with waiver of informed content. Main clinical data collected were sex, age, weight and height to calculate body mass index (BMI), albumin, C-reactive protein, smoking status, side, sarcopenia, presurgical treatments, reoperation, and complications within 30 days after pneumonectomy, classified as: lung complications, cardiac complications, other complications, and any complication. From an axial CT image at the level of L3, automatic segmentations were performed to calculate skeletal muscle area (SMA), skeletal muscle density, subcutaneous adipose tissue, and visceral adipose tissue. Skeletal muscle index was calculated as SMA/square height. Univariate and multivariate logistic regression analyses were performed to estimate the risk of any complication, both on the total population and in a by sex subgroup analysis. All tests were two tailed and considered significant at 5% level.

**Results:**

A total of 107 patients (84 men and 23 women) were included. Despite no significant differences in BMI, there were significant differences of body composition values in muscle and adipose tissue parameters between men and women, with women being significantly more sarcopenic than men (*p* = 0.002). Separate analyses for men and women showed that age and SMA were significantly associated with postoperative complications in men (*p* = 0.03 and 0.02, respectively).

**Conclusions:**

Body composition measurements extracted from routine CT may help in predicting complications after pneumonectomy, with men and women being different in quantity and distribution of muscle and fat, and men significantly more prone to postpneumonectomy complications with the increase of age and the decrease of skeletal muscle area.

## Introduction

In 2021, lung cancer is the second most frequent tumor in the USA in men and women, with 235,760 estimated new cases (119.100 in men and 116.600 in women) and the first cause of cancer-related deaths, with 131,880 estimated deaths (69.410 in men and 62470 in women) (1). According to stage and patient conditions, pneumonectomy may be considered for surgical resection of bulky and centrally located tumors, as well as for lung cancers that cannot be removed by lobectomy or smaller resections (2). The operative mortality of pneumonectomy has decreased over time, and in modern series, it approaches 5%–8%, although complication rates remain as high as 30%–40% (3, 4). This indicates that preoperative evaluation of candidates for pneumonectomy, currently based on a summative interpretation of multiple factors, including comorbities, evaluation of lung function (standard spirometry, perfusion lung scintigraphy), and cardiac performance (echocardiography, arterial blood gas analysis, cardiopulmonary exercise testing with respiratory oxygen uptake and carbon dioxide production measurement), remains unsatisfactory (5, 6).

On one hand, the assessment of body composition, indicating quantity and distribution of muscle and fat, has been associated with different clinical outcomes of cancer patients, such as tolerance to chemotherapy (7), survival, and postoperative outcomes, including lung cancer (8–11). On the other hand, lung cancer is being increasingly recognized as a heterogeneous disease in which gender plays a more critical role than previously appreciated in pathogenesis, diagnosis, and treatment (12).

Computed tomography (CT) represents a standard examination for cancer patients (13–15), and it is also considered a gold standard method for body composition assessment, because it makes it possible to measure the skeletal muscle area (SMA), at a desired anatomical level, and the fat infiltration of the muscle, measured by attenuation in skeletal muscle density (SMD), as well as the distribution of adipose tissue in visceral adipose tissue (VAT) and subcutaneous adipose tissue (SAT).

Previous studies have demonstrated that low muscle area may predict outcomes in patients undergoing pulmonary lobectomy (16, 17) and increased mortality after pneumonectomy (18). However, body composition differs between men and women, with men showing more lean mass, and women more fat mass (19). To the best of our knowledge, no studies have evaluated if different body composition parameters between men and women are associated with the advent of complications in lung cancer patients undergoing a major resection (pneumonectomy).

Therefore, the main purpose of this study was to assess if CT-derived body composition values, along with clinical characteristics, are associated with the risk of postsurgical complications in men and women who underwent pneumonectomy for lung cancer. A secondary exploratory purpose was to assess the correlation among body composition parameters, including muscle and fat measurements.

## Materials and Methods

### Patient Selection

The study population was retrospectively selected from a database of lung cancer patients who underwent pneumonectomy between Jan 2004 and Apr 2008. The ethics committee approved this retrospective study with waiver of informed content. Inclusion criteria consisted of: age ≥18 years; availability on the picture archiving and communication system (PACS) of a CT scan with iodinated contrast medium performed within 30 days before surgery. Exclusion criteria consisted of the following: technical problems on the CT images [such as from metallic prostheses (20)]; lack of the postcontrast acquisition; and documented refusal to the use of clinical data for research.

### Clinical Data Recording

The following clinical data were collected: sex, age at diagnosis, histological type, weight and height to calculate the body mass index (BMI), albumin, C-reactive protein, smoking status (never or former, smoker, unspecified), Glasgow prognostic store (GPS, 0,1 or 2), side (right, left), sarcopenia [as defined by Martin et al. (21)], presurgical treatments (no, yes), reoperation (no, yes), 30-day mortality (no, yes), and advent of complications occurring within 30 days after pneumonectomy, classified by a thoracic surgeon with 20 years of experience as: lung complications, cardiac complications, other complications, any complication (total complications). Forced expiratory level in the 1st second (FEV1) and diffusing capacity of the lung for carbon monoxide (DLCO) were also extracted from the clinical records at the time of surgery.

### CT Data Extraction

CT examinations were performed on different CT scanners at the same institution, and they were all available in digital format on local PACS. All the series used for extraction were acquired in the portal venous phase, after injection of contrast medium.

**Figure 1:**
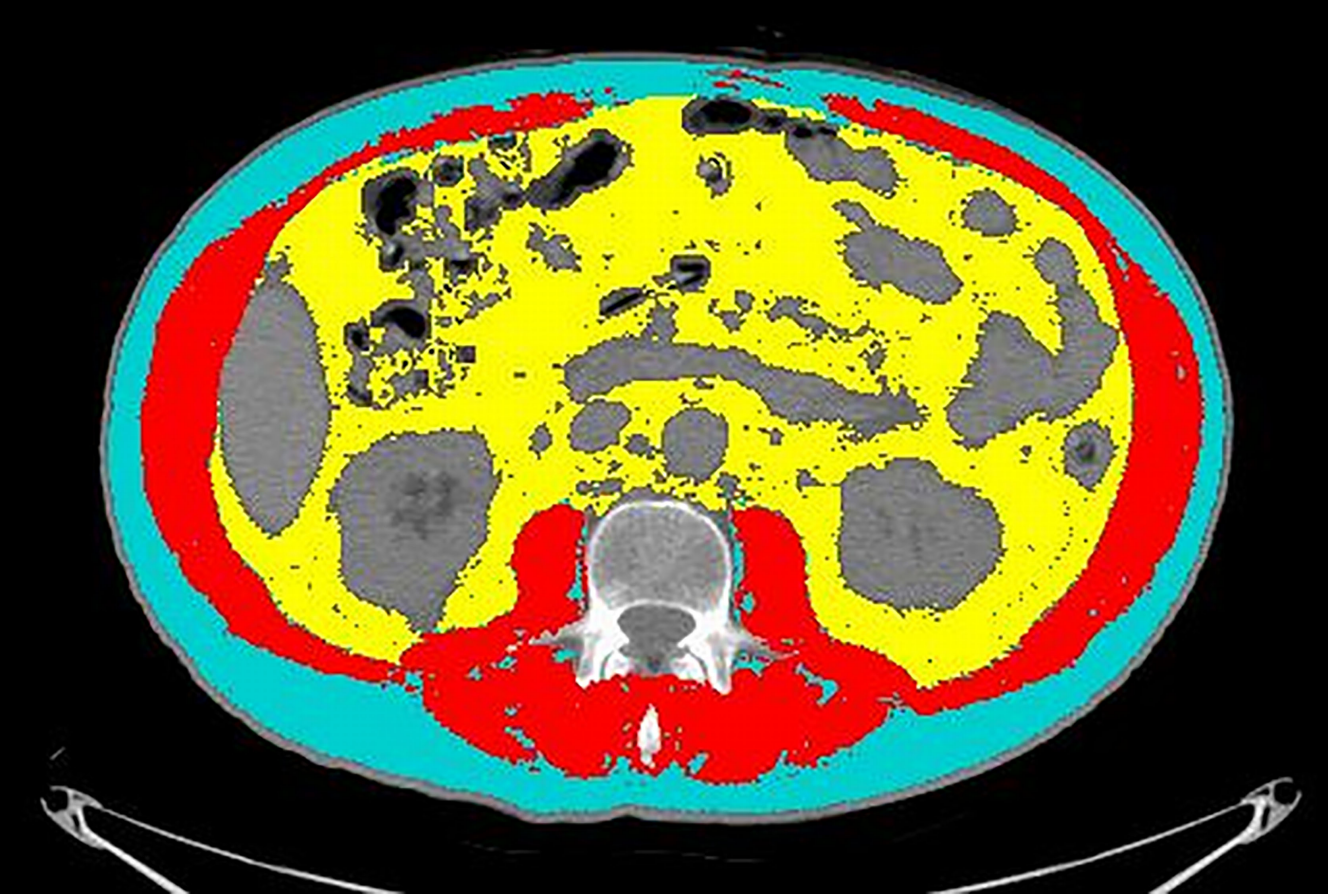
An example of segmentation of the CT image at the level of L3. Cyan indicates visceral adipose tissue (VAT) area; red indicates skeletal muscle area (SMA); yellow indicates visceral adipose tissue (VAT). Grey areas were excluded from measurements because they refer to abdominal organs (mainly liver, kidneys, and bowel).

An axial CT image at the level of L3 was selected and stored in digital imaging and communications in medicine format and then uploaded into the Slice-O-Matic software v 5.0 (Tomovision, Montreal, Canada). Segmentations of the muscle and fat were obtained by using the automated body composition analysis using the computed tomography image segmentation (ABACS) module, integrated in the software (**Figure 1**).

A radiologist with 16 years of experience double-checked the correspondence of the segmentation with the desired areas and, if needed, adjusted the segmentation using the semiautomatic tool. The following quantitative measurements were recorded: SMA (including the psoas, erector spinae, quadratus lumborum, transversus abdominis, external obliques, internal obliques, and rectus abdominis muscles) expressed in square centimeters (cm^2^); SMD expressed in Hounsfield units (HU); SAT expressed in cm^2^; and VAT expressed in cm^2^. The SMI was calculated by normalizing SMA by square height (m^2^), and reported as cm^2^/m^2^. The sex-specific cutoff to define sarcopenia was SMI <43 for men with BMI <24.9 and SMI <53 for men with BMI >25; whereas it was SMI <41 for women of any BMI (21).

### Statistical Analysis

Patients’ characteristics were summarized either by count and percent or mean, standard deviation (SD), and interquartile range (IQR) for categorical and continuous variables, respectively, by complication. Frequency distributions of the type of complications were tabulated as count and percentage. Summary statistics were computed and tabulated also by gender. Univariate and multivariate logistic regression analyses were performed to estimate the risk of any complication, both on the total population and in a by sex subgroup analysis. The results are expressed as odds ratios (OR) and tabulated alongside 95% confidence intervals (95% CI). A Pearson’s correlation analysis between muscle and adipose tissue parameters and a scatterplot matrix correlation were also produced. All tests were two-tailed and considered significant at the 5% level. All analyses were done using SAS 9.4 (N.C., Cary, USA).

## Results

One hundred and seven patients (84 men and 23 women), with a mean age of 62 ± 9.4 years, were included. All patients suffered from nonsmall cell lung cancer: 70 patients had adenocarcinoma and 37 patients had squamous cell carcinoma. Summary of patients’ characteristics, laboratory data, CT quantitative measurements of body composition, and outcomes by complications are provided for patients altogether in **Table 1**. Mean preoperative FEV 1 was 2.26 L (range 37.8–135 L); (81.70%); mean DLCO % was 84.2%; and mean perfusion of the affected lung: 39% (range 0%–56%). Pneumonectomy in the vast majority of cases was performed on nonpredominant lungs (perfusion <50% according to the perfusion scintigraphy). Type and number of complications are provided in **Supplementary Table S1**.

**Table 1:** Patient characteristics and outcomes by complication (any).

Characteristic	All patients (*N* = 107)	Any complication		*p*-value
		No (*N* = 53)	Yes (*N* = 54)	
**Sex**				
Male	84 (78.5)	41 (38.3)	43 (40.2)	
Female	23 (21.5)	12 (11.2)	11 (10.3)	0.82
**Age at surgery (years)**	62.0 (9.4)	59.6 (8.4)	64.3 (9.8)	**0.004**
**BMI (kg/m^2^)**	26.3 (4.8)	26.4 (4.9)	26.2 (4.7)	0.67
** Underweight (<20)**	10 (9.4)	6 (5.6)	4 (3.7)	
** Normal weight (20.0 to 24.9)**	34 (31.8)	16 (15.0)	18 (16.8)	
** Overweight/obese (≥25.0)**	63 (58.9)	31 (29.0)	32 (29.9)	0.84
**Albumin**	4.1 (0.4)	4.1 (0.4)	4.1 (0.3)	0.51
**C-reactive protein**	18.8 (2.0, 25.0)	20.5 (1.0, 26.0)	17.2 (2.0, 21.0)	0.93
**Smoking status**				
** Never or former**	5 (4.7)	6 (5.6)	5 (4.7)	
** Smoker**	48 (44.9)	46 (42.3)	48 (44.9)	0.88
** Unspecified**	1 (0.9)	1 (0.9)	1 (0.9)	
**Glasgow score**				
**0**	65 (60.7)	30 (28.0)	35 (32.7)	
**1**	35 (32.7)	19 (17.8)	16 (15.0)	0.70
**2**	7 (0.6)	4 (3.7)	3 (2.8)	
**Side**				
** Right**	46 (43.0)	23 (21.5)	23 (21.5)	
** Left**	61 (57.0)	30 (28.0)	31 (29.0)	1.00
**Presurgical treatments**	58 (54.2)	26 (24.3)	32 (29.9)	0.33
**Reoperation**	8 (7.5)	2 (1.9)	6 (5.6)	0.27
**30-Day mortality**	4 (3.7)	1 (0.9)	3 (2.8)	0.62
**Sarcopenia^a^**	58 (54.2)	26 (24.3)	32 (29.9)	0.33
**Skeletal muscle parameters**				
**SMA (cm^2^)**	133.8 (29.9)	138.6 (32.2)	129.1 (26.9)	0.10
**SMD (HU units)**	42.1 (7.8)	42.7 (7.9)	41.4 (7.7)	0.39
**SMI (cm^2^/m^2^)**	47.2 (9.8)	48.2 (10.2)	46.2 (9.4)	0.31
**SMA/SMD ratio**	3.3 (0.9)	3.4 (1.0)	3.2 (0.8)	0.39
**Adipose tissue**				
**VAT (cm^2^)**	144.2 (91.7)	146.9 (91.9)	141.5 (92.3)	0.86
**SAT (cm^2^)**	142.5 (73.9)	144.1 (70.0)	140.9 (78.2)	0.48

Statistics are mean (SD) for age, body mass index (BMI) (continuous), skeletal muscle parameters, adipose tissue, and albumin; mean (IQR) for C-reactive protein; and N (%) otherwise. ^a^See Martin et al. for definition. SMA, skeletal muscle area; SMD, skeletal muscle density; HU, Hounsfield units; SMI, skeletal muscle index; VAT, visceral adipose tissue; SAT, subcutaneous adipose tissue. Bold p-values are significant.

When the data are analyzed by sex, in spite of no significant differences in BMI, significant differences of body composition values in muscle parameters (SMA, SMI, SMA/SMD ratio) and adipose tissue parameters (VAT) between men and women become evident (**Table 2**), showing, for example, that female patients were significantly more sarcopenic than male patients (*p* = 0.002), with no significant difference among underweight, normal weight, and overweight/obese patients, compared with male patients.

**Table 2:** Comparison of patient characteristics by sex.

Characteristic	Sex			*p*-value	
	Male (*N* = 84)	Female (*N* = 23)			
**Age at surgery (years)**	62.5 (8.6)	59.9 (11.7)		0.41	
**BMI (kg/m^2^)**	26.7 (4.7)	24.6 (4.8)		0.05	
**Underweight (<20)**	6 (7.1)	4 (17.4)			
**Normal weight (20.0 to 24.9)**	25 (29.8)	9 (39.1)			
**Overweight/obese (≥25.0)**	53 (63.1)	10 (43.5)		0.15	
**Skeletal muscle parameters**				
**SMA (cm^2^)**	143.5 (25.7)	98.7 (12.9)		**<0.001**	
**SMD (HU units)**	42.2 (6.8)	41.4 (10.8)		0.74	
**SMI (cm^2^/m^2^)**	49.8 (9.1)	37.6 (5.4)		**<0.001**	
**SMA/SMD ratio**	3.4 (1.0)	3.2 (0.8)		**<0.001**	
**Adipose tissue**				
**VAT (cm^2^)**	163.6 (88.4)	73.2 (66.0)		<0.001	
**SAT (cm^2^)**	132.9 (59.2)	177.7 (107.1)		0.06	
**Albumin**	4.1 (0.4)	4.1 (0.4)		0.69	
**C-reactive protein**	20.3 (2.5, 25.5)	13.3 (0.0, 9.0)		0.11	
**Smoking status**				
**Never or former**	3 (3.6)	8 (34.8)			
**Smoker**	79 (94.1)	15 (65.2)		**0.002**	
**Unspecified**	2 (2.4)	0			
**Glasgow score**				
**0**	47 (56.0)	18 (78.3)			
**1**	31 (36.9)	4 (17.4)		0.15	
**2**	6 (7.1)	1 (4.4)			
**Side**				
**Right**	36 (42.9)	10 (43.5)			
**Left**	48 (57.1)	13 (56.5)		1.00	
**Sarcopenia^a^**	39 (46.4)	19 (82.6)		**0.002**	
**Presurgical treatments**	45 (53.6)	13 (56.5)		0.82	
**Reoperation**	8 (9.5)	0		0.20	
**30-Day mortality**	1 (1.9)	3 (5.6)		1.00	

Statistics are mean (SD) for age, BMI (continuous), skeletal muscle parameters, adipose tissue, and albumin; mean (IQR) for C-reactive protein; and N (column %) otherwise. ^a^See Martin et al. for definition. SMA, skeletal muscle area; SMD, skeletal muscle density; HU, Hounsfield units; SMI, skeletal muscle index; VAT, visceral adipose tissue; SAT, subcutaneous adipose tissue. Bold p-values are significant.

Separate analyses for men (**Table 3**) and women (**Table 4**) showed that age and SMA were significantly associated with postoperative complications in men. More specifically, univariate risk estimates analysis showed a significant increased risk of complications (*p* = 0.03) in men with higher age at surgery (OR = 1.04, 95% CI: 1.79) (**Table 5**), and with lower SMA (*p* = 0.02), with a decreased risk for each 10 cm^2^ increase of SMA (OR = 0.80, 95% CI: 0.66, 0.96), which was borderline significant at the multivariate analysis (*p* = 0.05).

**Table 3:** Patient characteristics and outcomes by complication (any) in male patients.

Characteristic	Male patients (*N* = 84)	Any complication		*p*-value
		No (*N* = 41)	Yes (*N* = 43)	
**Age at surgery (years)**	62.5 (8.6)	60.3 (7.7)	64.6 (8.9)	**0.02**
**BMI (kg/m^2^)**	26.7 (4.7)	27.1 (4.9)	26.4 (4.6)	0.43
** Underweight (<20)**	6 (7.1)	3 (3.6)	4 (3.6)	
** Normal weight (20.0 to 24.9)**	25 (29.8)	12 (14.3)	13 (15.5)	
** Overweight/Obese (≥25.0)**	53 (63.1)	26 (31.0)	27 (32.1)	1.00
**Skeletal muscle parameters**				
** SMA (cm^2^)**	143.5 (25.7)	150.4 (26.1)	136.8 (23.8)	**0.01**
** SMD (HU units)**	42.2 (6.8)	42.8 (6.2)	41.6 (7.3)	0.42
** SMI (cm^2^/m^2^)**	49.8 (9.1)	51.5 (9.0)	48.3 (9.0)	0.09
** SMA/SMD ratio**	3.4 (1.0)	3.6 (0.8)	3.4 (0.7)	0.20
**Adipose tissue**				
** VAT (cm^2^)**	163.6 (88.4)	167.3 (86.4)	160.1 (91.0)	0.71
** SAT (cm^2^)**	132.9 (59.2)	137.5 (59.1)	128.4 (59.6)	0.48
**Albumin**	4.1 (0.4)	4.1 (0.4)	4.1 (0.3)	0.37
**C-reactive protein**	20.3 (2.5, 25.5)	22.1 (3.0, 26.0)	18.6 (2.0, 25.0)	0.83
**Smoking status**				
** Never or former**	3 (3.6)	2 (2.4)	1 (1.2)	
** Smoker**	79 (94.1)	38 (45.2)	41 (48.8)	0.80
** Unspecified**	2 (2.4)	1 (1.2)	1 (1.2)	
**Glasgow score**				
** 0**	47 (56.0)	21 (25.0)	26 (31.0)	
** 1**	31 (36.9)	17 (20.2)	14 (16.7)	0.70
** 2**	6 (7.1)	3 (3.6)	3 (3.6)	
**Side**				
** Right**	36 (42.9)	15 (17.9)	21 (25.0)	
** Left**	48 (57.1)	26 (31.0)	22 (26.2)	0.28
**Sarcopenia^a^**	39 (46.4)	15 (17.9)	24 (28.6)	0.09
**Presurgical treatments**	45 (53.6)	20 (23.8)	25 (29.8)	0.51
**Reoperation**	8 (9.5)	2 (2.4)	6 (7.1)	0.27
**30-Day mortality**	3 (3.6)	0	3 (3.6)	0.24

Statistics are mean (SD) for age, body mass index (BMI) (continuous), skeletal muscle parameters, adipose tissue, and albumin; mean (IQR) for C-reactive protein; and N (%) otherwise. ^a^See Martin et al. for definition. SMA, skeletal muscle area; SMD, skeletal muscle density; HU, Hounsfield units; SMI, skeletal muscle index; VAT, visceral adipose tissue; SAT, subcutaneous adipose tissue. Bold p-values are significant.

**Table 4:** Patient characteristics and outcomes by complication (any) in female patients.

Characteristic	Female patients (*N* = 23)	Any complication		*p*-value
		No (*N* = 12)	Yes (*N* = 11)	
**Age at surgery (years)**	59.9 (11.7)	57.1 (10.3)	62.9 (12.9)	0.25
**BMI (kg/m^2^)**	24.6 (4.8)	23.7 (4.5)	25.5 (5.3)	0.41
** Underweight (<20)**	4 (17.4)	3 (13.0)	1 (4.4)	
** Normal weight (20.0 to 24.9)**	9 (39.1)	4 (17.4)	5 (21.7)	
** Overweight/obese (≥25.0)**	10 (43.5)	5 (21.7)	5 (21.7)	0.74
**Skeletal muscle parameters**				
** SMA (cm^2^)**	98.7 (12.9)	98.2 (11.5)	99.2 (14.9)	0.85
** SMD (HU units)**	41.4 (10.8)	42.3 (12.3)	40.5 (9.2)	0.70
** SMI (cm^2^/m^2^)**	37.6 (5.4)	37.0 4.5)	38.3 (6.4)	0.57
** SMA/SMD ratio**	2.6 (0.9)	2.6 (1.2)	2.6 (0.6)	0.29
**Adipose tissue**				
** VAT (cm^2^)**	73.2 (66.0)	77.2 (77.0)	68.8 (55.0)	0.90
** SAT (cm^2^)**	177.7 (107.1)	166.7 (98.7)	189.8 (119.2)	0.98
**Albumin**	4.1 (0.4)	4.2 (0.4)	4.1 (0.3)	0.85
**C-reactive protein**	13.3 (0.0, 9.0)	15.0 (0.5, 19.5)	11.5 (0.0, 9.0)	0.98
**Smoking status**				
** Never or former**	8 (34.8)	4 (17.4)	4 (17.4)	
** Smoker**	15 (65.2)	8 (34.8)	7 (30.4)	1.00
**Glasgow score**				
** 0**	18 (78.3)	9 (39.1)	9 (39.1)	
** 1**	4 (17.4)	2 (8.7)	2 (8.7)	1.00
** 2**	1 (4.4)	1 (4.4)	0	
**Side**				
** Right**	10 (43.5)	8 (34.8)	2 (8.7)	
** Left**	13 (56.5)	4 (14.4)	9 (39.1)	**0.04**
**Sarcopenia^a^**	19 (82.6)	11 (47.8)	8 (34.8)	0.32
**Presurgical treatments**	13 (56.5)	6 (26.1)	7 (30.4)	0.68
**Reoperation**	0	0	0	–
**30-Day mortality**	1 (4.4)	1 (4.4)	0	1.00

Statistics are mean (SD) for age, body mass index (BMI)(continuous), skeletal muscle parameters, adipose tissue, and albumin; mean (IQR) for C-reactive protein, and N (%) otherwise. ^a^See Martin et al. for definition. SMA, skeletal muscle area; SMD, skeletal muscle density. HU, Hounsfield units; SMI, skeletal muscle index; VAT, visceral adipose tissue; SAT, subcutaneous adipose tissue. Bold p-values are significant.

**Table 5:** Univariate and multivariate risk estimates analysis for any complication, by sex.

Sex	Univariate OR (95% CI)	*p*-value	Multivariate OR (95% CI)	*p*-value
**Male**				
** Age at surgery**	1.36 (1.04,1.79)^a^	**0.03**	1.05 (0.99,1.11)	0.07
** SMA (cm^2^)**	0.80 (0.66,0.96)^b^	**0.02**	0.91 (0.83,1.00)	0.05
** SMD (HU units)**	0.77 (0.40,1.46)^b^	0.42	–	–
** SMI (cm^2^/m^2^)**	0.67 (0.41,1.10)^b^	0.11	–	–
** SMA/SMD ratio**	0.67 (0.37,1.22)^c^	0.19	–	–
** VAT (cm^2^)**	0.99 (0.94,1.04)*^b^*	0.71	–	–
**Female**				
** Age at surgery**	1.26 (0.86,1.86)^a^	0.23	–	–
** SMA (cm^2^)**	1.07 (0.56,2.05)^b^	0.84	–	–
** SMD (HU units)**	0.85 (0.39,1.86)^b^	0.68	–	–
** SMI (cm^2^/m^2^)**	1.64 (0.33,8.13)^b^	0.54	–	–
** SMA/SMD ratio**	0.93 (0.38,2.26)^c^	0.87	–	–
** VAT (cm^2^)**	0.98 (0.86,1.11)^b^	0.76	–	–

^a^By 5-year increase. ^b^By 10-unit increase. ^c^By 1-unit increase. SMA, SMI and SMA/SMD ratio; OR, odds ratio; CI, confidence interval; SMA, skeletal muscle area; SMD, skeletal muscle density; HU, Hounsfield units; SMI, skeletal muscle index; VAT, visceral adipose tissue. Bold p-values are significant.

**Figure 2:**
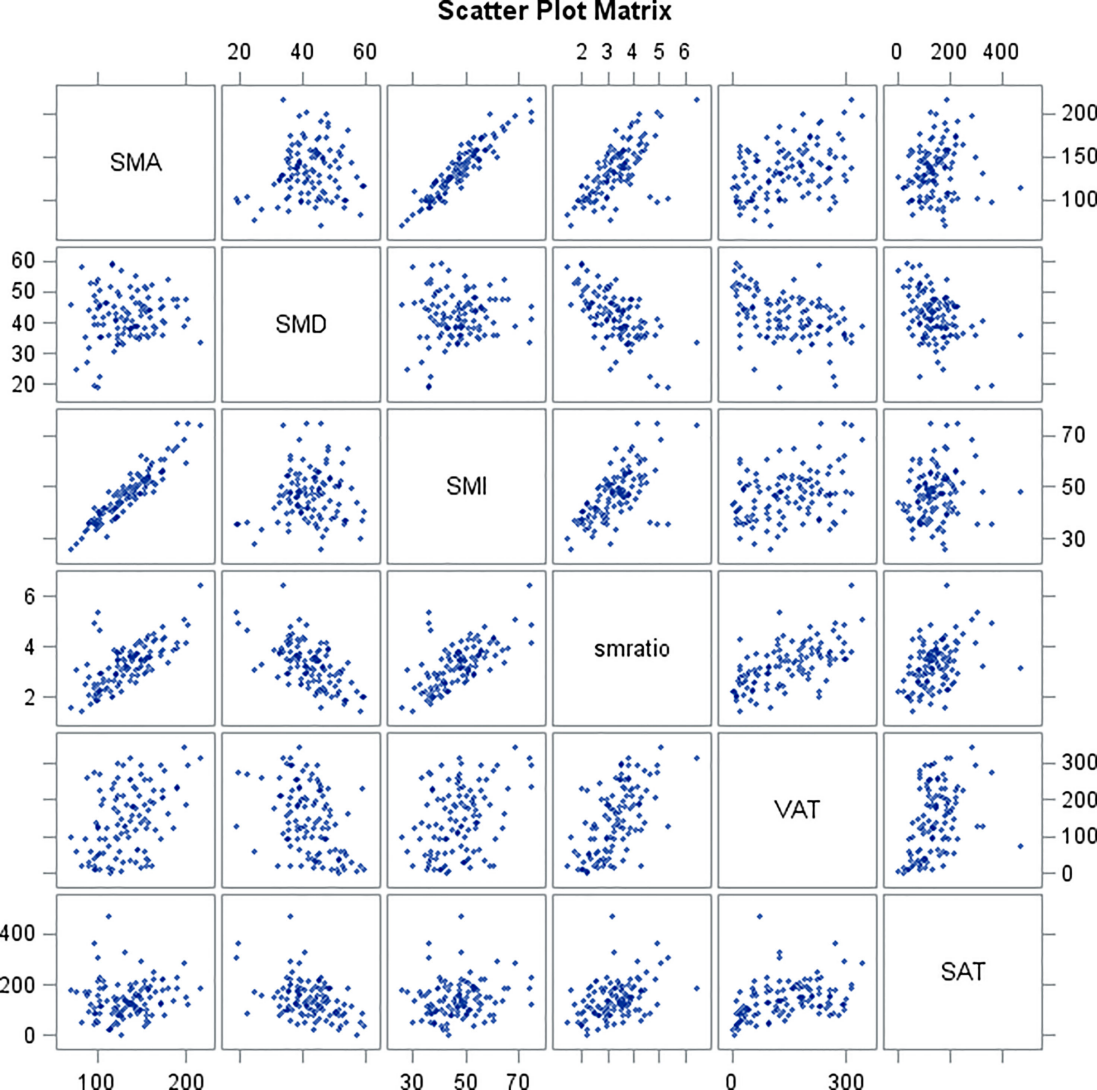
Scatterplot matrix of skeletal muscle and adipose tissue parameters.

**Table 6** and scatter plot matrix (**Figure 2**) show correlations between skeletal muscle parameters (SMA, SMD, SMI, SMA/SMD) and adipose tissue parameters (VAT, SAT), where SMA shows no correlation with SMD and SAT, SMD shows no correlation with SMI, and SAT shows no correlation with SMI.

**Table 6:** Pearson’s correlations between skeletal muscle parameters and adipose tissue parameters.

	SMD	SMI	SMA/SMD ratio	VAT	SAT
**SMA**	0.10; *p* = 0.28	0.93; ***p* < 0.001**	0.68; ***p* < 0.001**	0.42; ***p* < 0.001**	0.13; ***p* = 0.19**
**SMD**		0.07; *p* = 0.45	−0.62; ***p* < 0.001**	−0.42; ***p* < 0.001**	−0.43; ***p* < 0.001**
**SMI**			0.65; ***p* < 0.001**	0.36; ***p* < 0.001**	0.17; ***p* = 0.09**
**SMA/SMD ratio**				0.61; ***p* < 0.001**	0.40; ***p* < 0.001**
**VAT**					0.37; ***p* < 0.001**

## Discussion

The role of sex in the development, treatment, and prognosis of many malignancies has been so far underappreciated and understudied (12). In 1993, the National Institute of Health Revitalization Act required inclusion of women in their funded trials, but it was not until October 2014 that a formal policy was established requiring a balanced approach to gender analysis in research studies (22). As a result, knowledge regarding sex differences in oncologic diseases is severely lagging. Concerning comparisons of lung cancer patients, numerous studies have shown that men show lower overall survival and disease-free survival regardless of histology, stage, or treatment, compared with women (23–25). A meta-analysis of pooled hazard ratios of 86,800 patients revealed higher survival rates for women with nonsmall-cell lung cancer both in univariate and multivariate analyses (23).

Surgical resection is generally considered the standard of care for the majority of early-stage NSCLC, and used in combination with chemotherapy, radiation, targeted therapy, and immunotherapy for more advanced disease. Despite this, it has been reported that women undergo appropriate surgical intervention for NSCLC at lower rates compared with men (26, 27) and that they are more likely to undergo a more limited surgical resection compared with men (28).

In a study comparing men and women undergoing lung cancer resections, a univariate analysis of individual postoperative complications showed that sex was a significant predictor in nearly all events examined, including pulmonary, cardiac, neurologic, hematologic, infectious, and gastrointestinal categories (29). In their study, Tong et al. demonstrated that women and men undergoing surgery for lung cancer differ with regard to preoperative characteristics and comorbidities (29). Accordingly, in our cohort, we found significant differences in body composition distribution between men and women. Specifically, we demonstrated no significant difference in BMI distribution between the two sexes, but significant differences in muscle and adipose tissue distribution, with men exhibiting higher values of SMA and SMI than women, as well as higher values of VAT than women. Furthermore, despite no significant differences in BMI between the two groups, women were more prone to sarcopenia than men.

These differences between men and women support a separate sex-related analysis.

In our cohort, by analyzing the association of body composition measurements and postpneumonectomy complications, among 107 patients who underwent pneumonectomy for lung cancer, we found significant differences in muscle and adipose tissue distribution in men and women, with a significant correlation of age and SMA with postsurgical complications in men.

Association between low muscle mass and poor outcomes in terms of overall survival (OS) was demonstrated in lung cancer patients (18, 30, 31), as well as in other cancers (32). However, it is well known that if OS is considered the main outcome, many comorbidities associated with body composition (including obesity-related and cachexia-related pathologies) may have a role in leading the patient to death.

Enhanced recovery after surgery is well established in specialties such as colorectal surgery. It is achieved through the introduction of multiple evidence-based perioperative measures that aim to reduce postoperative organ dysfunction while facilitating recovery. Evidence from abdominal surgery suggests that routine pre- and/or postoperative oral nutritional supplements reduce postoperative weight loss, improve nutritional status and muscle strength, and reduce complication rates (33). In thoracic surgery, preoperative interventions may include nutritional assessment and treatment, anemia correction, and smoking cessation. With this regard, guidelines of the European Society of Thoracic Surgeons (ESTS) recommend to screen patients preoperatively for nutritional status and weight loss; then, if deemed at risk, patients should be given active nutritional support, including oral nutritional supplements (34). According to these recommendations, our results support a preoperative evaluation of nutritional status of the patient, with the possibility to improve it, through nutritional supplements, if needed. In this study, we concentrated our attention on the advent of complications after pneumonectomy, because the complication rates of this surgery are still high (30%–40%), accounting for an insufficient preoperative evaluation (5, 6).

In 1999, the Veterans Affairs National Surgical Quality Improvement Program published the results of data obtained prospectively on 3516 consecutive patients who had undergone major lung resection across 123 medical centers over almost 4 years. Although the authors analyzed the largest dataset available at that time with the aim of creating a risk-adjusted model for selection of patients for lung resection, their data lacked pulmonary function testing and were heavily biased toward a male population, leading to doubts about the possibility of generalizing the results (35, 36).

Since then, many authors have proposed models to predict the risk of complications after lung surgery, such as a cardiopulmonary risk index combining the presence of cardiac variables, with pulmonary variables (37); the physiologic and operative severity score for enumeration of mortality and morbidity combining a 12-factor physiologic score with a 6-factor operative severity score (38); a score including the percentage of forced expiratory level, age, and diffusing capacity of the lung for carbon monoxide (DLCO) (39). More recently, a combined outcome index score included 30-day mortality, 30-day cardiopulmonary morbidity, unplanned admission to the intensive care unit, and prolonged hospital stay, including as covariates age, predicted postoperative FEV1, cardiac comorbidities, pneumonectomy, and performance status (40). Unfortunately, only some of the abovementioned risk models include sex as a covariate, and none of them includes body composition measurements. Body composition may be an important feature in many clinical settings as it is associated with efficacy and toxicity of therapies, with patient functional status as well as with surgical complication rates and survival (41–43), also in lung cancer patients (44).

Therefore, an open question is whether an accurate prediction of post-pneumonectomy complications is still lacking because of the missed inclusion of potentially important features, such as body composition measurements.

With a focus on post-surgical complications, not including pneumonectomy, Suzuki et al., demonstrated higher complications associated with low SMA in 90 patients with stage I lung cancer who underwent wedge resection, segmentectomy or lobectomy (30). Similarly, in 135 NSCLC patients, Fintelmann demonstrated that SMA was significantly associated with postlobectomy complications, hospital length of stay, and hospital readmission (45). In a larger cohort of 958 patients, best demonstrated that muscle (using 30 cm^2^ increments) was an independent predictor of length of stay, any postoperative complications and postoperative respiratory complications (46). In a cohort more similar to ours, Hervochon et al. evaluated patients undergoing pneumonectomy, measuring psoas muscle area at the L3 level, and showed associations between body composition and OS, where SMA was not an independent predictor of OS; unfortunately, their study was not designed to evaluate postoperative complications and furthermore they did not include sex and age in their analysis (17). Madariaga et al. evaluated 130 patients who underwent pneumonectomy and they demonstrated that patients with high SMA (measured at the level of the eighth and twelfth vertebral bodies) experienced fewer overall and cardiopulmonary complications, and fewer readmissions (5). Furthermore, in their cohort, men and women showed a significant different distribution of SMA, although further evaluations included patients in the lowest and upper quartile of SMA, instead of a division by sex.

Our results are concordant with Madariaga et al. (5), although our evaluation of SMA was made at the level of L3, thus suggesting that body composition is important and correlated with postpneumonectomy complications at the thoracic as well as the lumbar level. Furthermore, our findings suggest that CT images contain relevant information with the potential to improve existing risk prediction models. CT scans are routinely performed in lung cancer patients for pretreatment staging, as well as for follow-up under or after treatment (47), therefore this technique may offer an opportunistic evaluation of the body composition status of the patients, without impairing their standard workflow. With the use of specific software, it is possible to extract the quantitative measurements of SMA, SAT, SMD, and VAT in a single evaluation. Since many body composition measurements are independent one from the other, as demonstrated by our secondary analysis, it is advisable to extract all of them from a single axial CT image.

This study has some limitations. First, the segmentation of the body composition measurements on CT images was made by using an automatic tool. It is known that there is a borderline balance between the precision of semi-automatic segmentations, less time-efficient as they take longer to perform, and fast automatic segmentations, sometimes lacking precision. However, in order to overcome this limitation, the radiologist who performed the segmentations always double-checked the automatic segmentation and, if not satisfied, did it with the semi-automatic tool. Second, we assessed SMA and sarcopenia according to the mentioned definitions, with no evaluation of muscle strength, frequently included in the definition of sarcopenia (48), but in a retrospective evaluation, these data were not available. However, given these promising results and the suggested importance of body composition, assessment of sarcopenia could include muscle strength in prospective future studies. Third, we found a significant association between SMA and complications in men, despite a higher proportion of sarcopenia in women. This result suggests that women have some type of compensation to this difference, but we did not go further in this evaluation that, in our opinion, may deserve a dedicated study. Lastly, we found significant differences between men and women, but the population was not equally distributed (84 men and 23 women) because the selection was retrospective and in the period selected, we found only 23 women. Therefore, further studies will be needed to confirm our results.

In conclusion, our study demonstrates that body composition measurements extracted from routine CT may help in predicting complications after pneumonectomy, men and women being different in quantity and distribution of muscle and fat, and men significantly more prone to postpneumonectomy complications with the increase of age and the decrease of skeletal muscle area.

## Data Availability Statement

The datasets presented in this article are not readily available because approval for sharing the database was not requested to the ethics committee. Requests to access the datasets should be directed to francesco.petrella@ieo.it.

## Ethics Statement

The studies involving human participants were reviewed and approved by the IEO-CCM ethics committee. Written informed consent for participation was not required for this study in accordance with the national legislation and the institutional requirements.

## Author Contributions

Conception and design: all authors. Administrative support: SR and FP. Provision of study materials or patients: FP, CB, LB, AC, SM, and LS. Collection and assembly of data: SR, FP, DR, and GR. Data analysis and interpretation: SR, FP, and DR. Manuscript writing: all authors. Final approval of manuscript: all authors. All authors listed have made a substantial, direct, and intellectual contribution to the work and approved it for publication.

## Conflict of Interest

The authors declare that the research was conducted in the absence of any commercial or financial relationships that could be construed as a potential conflict of interest.

## Publisher’s Note

All claims expressed in this article are solely those of the authors and do not necessarily represent those of their affiliated organizations, or those of the publisher, the editors and the reviewers. Any product that may be evaluated in this article, or claim that may be made by its manufacturer, is not guaranteed or endorsed by the publisher.
