# Supplementary material for: CT-Derived Body Composition Values and Complications After Pneumonectomy in Lung Cancer Patients: Time for a Sex-Related Analysis?
Source: Front Oncol. 2022 Mar 15;12:826058. doi: 10.3389/fonc.2022.826058 (PMC8964946; doi:10.3389/fonc.2022.826058)
Supplement: Supplementary file 1 [file Table_1.docx]

**Supplementary Table 1.** Frequency distribution of complications

|  | **N (%)** |
| --- | --- |
| **No complications** | 53 (49.6) |
| **Lung complications** | 6 (6.5) |
| **Cardiac complications** | 10 (9.4) |
| **Other complications** | 20 (18.7) |
| **Lung complications + Other complications** | 3 (2.8) |
| **Lung complications + Cardiac complications + Other complications** | 4 (3.7) |
| **Cardiac complications + Other complications** | 11 (10.3) |
| **Any complication** | 54 (50.5) |
